# The psychometric characteristics of the revised depression attitude questionnaire (R-DAQ) in Pakistani medical practitioners: a cross-sectional study of doctors in Lahore

**DOI:** 10.1186/s13104-017-2652-3

**Published:** 2017-07-27

**Authors:** Mark Haddad, Ahmed Waqas, Ahmed Bashir Sukhera, Asad Zaman Tarar

**Affiliations:** 1 Centre for Mental Health Research; School of Health Sciences, City University London, Northampton Square, London, EC1V 0HB UK; 2 East London NHS Foundation Trust, London, UK; 3 CMH Lahore Medical College and Institute of Dentistry, Lahore, Pakistan

**Keywords:** Depression, Questionnaire, Attitudes, Psychometrics, Factor analysis

## Abstract

**Background:**

Depression is common mental health problem and leading contributor to the global burden of disease. The attitudes and beliefs of the public and of health professionals influence social acceptance and affect the esteem and help-seeking of people experiencing mental health problems. The attitudes of clinicians are particularly relevant to their role in accurately recognising and providing appropriate support and management of depression. This study examines the characteristics of the revised depression attitude questionnaire (R-DAQ) with doctors working in healthcare settings in Lahore, Pakistan.

**Methods:**

A cross-sectional survey was conducted in 2015 using the revised depression attitude questionnaire (R-DAQ). A convenience sample of 700 medical practitioners based in six hospitals in Lahore was approached to participate in the survey. The R-DAQ structure was examined using Parallel Analysis from polychoric correlations. Unweighted least squares analysis (ULSA) was used for factor extraction. Model fit was estimated using goodness-of-fit indices and the root mean square of standardized residuals (RMSR), and internal consistency reliability for the overall scale and subscales was assessed using reliability estimates based on Mislevy and Bock (BILOG 3 Item analysis and test scoring with binary logistic models. Mooresville: Scientific Software, 55) and the McDonald’s Omega statistic. Findings using this approach were compared with principal axis factor analysis based on Pearson correlation matrix.

**Results:**

601 (86%) of the doctors approached consented to participate in the study. Exploratory factor analysis of R-DAQ scale responses demonstrated the same 3-factor structure as in the UK development study, though analyses indicated removal of 7 of the 22 items because of weak loading or poor model fit. The 3 factor solution accounted for 49.8% of the common variance. Scale reliability and internal consistency were adequate: total scale standardised alpha was 0.694; subscale reliability for professional confidence was 0.732, therapeutic optimism/pessimism was 0.638, and generalist perspective was 0.769.

**Conclusions:**

The R-DAQ was developed with a predominantly UK-based sample of health professionals. This study indicates that this scale functions adequately and provides a valid measure of depression attitudes for medical practitioners in Pakistan, with the same factor structure as in the scale development sample. However, optimal scale function necessitated removal of several items, with a 15-item scale enabling the most parsimonious factor solution for this population.

## Background

Mental health problems are a major cause of disability throughout the world: the World Health Organization (WHO) global burden of diseases study (2013) indicates that nearly a quarter (21.2%) of global years lived with disability (YLDs) are caused by mental and substance abuse disorders [1]. Depressive disorder carries the heaviest burden of all the mental disorders, and is the second leading cause of global disability by YLD (after low back pain). It occurs in all world regions and affects people of all ages, accounting for 8.2% of global YLDs [2].

Depression is two to three times more common in people with long-term medical conditions such as asthma, cardiovascular disease, cancer, and diabetes [3], with WHO World Health Survey findings, based on 245,000 people in 60 countries, indicating it occurs in 9–18% of people with such conditions [4]. It adversely affects the course and prognosis of these illnesses, compounding the disability and impaired life quality that people experience. Because of its relatively high prevalence and the extent of its comorbid association with medical conditions, depression is frequently encountered in primary care and general medical settings.

Negative public attitudes to mental health problems are widely held and add to the difficulties experienced by people with these problems [5]. Stigma about mental health is based on misconceptions about the nature of these health problems and inhibits disclosure and help-seeking [6]. Research indicates that critical and stigmatising views are held to varying degrees by health professionals as well as the public [7], and that they are evident in western and non-western societies [8]. Health professionals’ attitudes to mental illness may incorporate some of the stereotypes and misunderstandings pervasive in society, and may also reflect inadequate or poorly designed training. Patients highly value health professionals’ interpersonal skills, and for people with depression this area has been identified as especially important [9]: listening, showing understanding, approaching patients as individuals, and making patients feel comfortable are regarded as most important factors in medical consultations. Similarly, being able to be involved in treatment decisions appears to be associated with a greater probability of receiving evidence-based depression treatment and symptom improvement [10]. One reason for examining clinicians’ attitudes to depression is because of this effect on patient help-seeking and involvement in care.

Clinicians’ attitudes are also likely to influence their recognition of conditions and subsequent clinical behaviour. The detection of depression in primary care and other non-psychiatric settings is often problematic, in part because patients typically present with a combination of physical, psychological, and social problems [11], and there is good evidence that around half of primary care cases are missed at initial consultation. A meta-analysis of 41 studies examining general practitioner’s (GP’s) clinical ability to detect depression (defined by diagnostic interview) revealed only 47% of depression cases were correctly identified [12], with substantial differences in detection accuracy between nations [13]. It is likely that clinicians’ attitudes to depression contribute to its correct identification, as well as to assessment and management approaches [14, 15].

Research concerning the views of health professionals towards mental illness in non-western nations is relatively limited, and many of the studies in this area have either explored attitudes and stigma related to a range of mental health problems (rather than depression in particular) [16, 17], or been based on samples of medical students (rather than practicing clinicians) [18–21], or used attitude measures of weak or uncertain validity and reliability [22–24].

This study reports the first use of the revised depression attitude questionnaire (R-DAQ) [25] with doctors working in Lahore, Pakistan. The R-DAQ is a revised version of a widely used scale, the depression attitude questionnaire (DAQ), that was developed with GPs in the UK in the early 1990s [26]. The revised version [25] was developed to address weaknesses in the DAQ, and its construction involved a structured consultation technique incorporating the views of researchers and clinicians from the USA, UK and several other European countries and initial testing with a sample of 1193 health professionals (largely nurses and from the UK).

This study aimed to examine the psychometric characteristics of the R-DAQ when used by medical practitioners in Pakistan by comparing the factor structure and measures of internal consistency with the findings of the UK development study, as well as between selected (‘known’) groups within this sample based on involvement in psychiatry training or professional development. The findings reported in this paper compliment the analysis of relationships between participant characteristics including casual attributions for depression, and depression attitudes, that are fully provided in a further paper [27].

## Methods

This was a cross-sectional study using a self-complete survey questionnaire distributed to a convenience sample of medical practitioners based at six hospitals in Lahore, Pakistan.

### Setting

Pakistan is a South Asian nation with population of 185.1 million; based upon World Bank criteria it is a low income nation and it ranks is 147 out of 188 countries and territories according to the human development index (HDI), a summary measure of healthy life expectancy, standard of living and access to knowledge. Life expectancy from birth (66.2 years) is lower than for the South Asian region (68.4 years) and world (71.5 years) [28]. Health expenditure in Pakistan is estimated by the World Bank [29] as 2.8% of gross domestic product (GDP), and 0.4% of health expenditure is devoted to mental health [30].

Lahore is the second largest city in Pakistan, with an estimated population of 7.6 million. This survey was administered at six hospitals in Lahore: Mayo hospital, Jinnah hospital, combined military hospital and services hospital, doctors hospital, Lahore general hospital, between June 2015 and September 2015.

### Measures

A single self-report questionnaire was used consisting of the R- DAQ scale [25]; questions concerning participant demographics and medical specialism education and training; and items relating to the possible causes of depression based on a format previously used in a study of university students views about mental illness [20]. Findings concerning the relationships between casual attributions for depression and attitudes measured by the R-DAQ are reported in a further paper [27].

The R-DAQ is a 22-item scale derived from the 20-item DAQ [26]. The original DAQ has been used in studies conducted in Europe [31], Australia [32], Japan [33] and elsewhere [34], with GPs and other health professionals including nurses [35–37] and pharmacists [38]. Despite its extensive use, the DAQ has been identified as having psychometric weaknesses, as well problems in relation to its complex wording and its development with a specific professional group based only in Britain which potentially limited is suitability for use with the wider professional workforce involved in depression identification, support and management. The revised version was developed following a pooled analysis of study findings [31] and a Delphi consensus study involving experts from UK, the USA, Australia, Belgium, Finland, Estonia, and Italy [25]. The 22-item R-DAQ incorporates 9 items from the original 20-item DAQ scale, together with additional items derived and adapted from other mental illness attitude measures including the defeat depression campaign Mori poll questionnaire [39], European alliance against depression (EAAD) instruments [40], the survey derived from the community attitudes toward the mentally Ill (CAMI) measure [41] used within the research surveys of Great Britain (RSGB) Omnibus [42], as well as items proposed and reviewed by the Delphi panel. The scale items are attitude statements with response options noting level of agreement between ‘strongly agree’ and ‘strongly disagree’, scored with a five-point Likert scale.

The R-DAQ initial study involved a sample of 1193 health professionals but these were largely nurses and almost entirely from the UK. In this development study psychometric adequacy was evident: the total scale internal consistency was 0.84, and there was clear construct validity, easy readability, and minimal floor and ceiling effects. Three sub-scales were evident in the R-DAQ development sample concerning respondents’ attitudes about ‘*professional confidence in depression care*’, ‘*therapeutic optimism about depression’* and ‘*generalist perspective about depression occurrence, recognition and management*’.

### Procedure

Ethical approval for the study was sought and provided by the ethical review committee of CMH Lahore medical college, Lahore, Pakistan. Convenience sampling was employed and all available medical practitioners, excluding any who had formal diploma or fellowship training in psychiatry or were practicing as psychiatrists, were approached by four medical students who visited the sites on several occasions over a 3-month period. These researchers provided the participants with study information, a consent form and the survey questionnaire, and all potential participants were ensured anonymity and informed that the reporting of survey responses would be not enable identification of any individual.

The R-DAQ did not require translation as English is the second official language of Pakistan and widely spoken, especially among professionals, and is used as the medium of instruction in all the medical schools. The questionnaire was pilot tested with six Pakistani medical students to ensure that the phrasing, terminology, layout, and time taken to complete the survey were understandable and appropriate to the target population of Pakistani medical practitioners. Some minor modifications of the demographic items were required, and there were no issues with the readability of the R-DAQ (and examination established the Flesch–Kincaid grade level was 9.4, indicating it to be understandable to a typical 14–15 year-old student).

### Analysis

The survey sample size was determined in relation to exploratory factor analysis (EFA) of the R-DAQ: calculation was based on the pattern of communalities in the variables being relatively wide, the expected ratio of variables to factors being between 4 and 5, and an expected three-factor solution. These considerations indicated that a sample of between 200 and 350 was necessary [43]. We anticipated, on the basis of response rates in similar studies conducted within a similar population [20, 21], that a total of 700 medical practitioners would need to be contacted to provide our required sample.

The study data were analysed with the FACTOR (10.03.01) programme [44], using unweighted least squares (ULS) with promin rotation (an oblique method). The freely available FACTOR programme enables the factor analysis to be computed from a polychoric dispersion matrix, which is of particular value in regard to the common situation of analysing ordinal data such as derived from Likert-type scales. EFA based on a dispersion matrix using Pearson correlations (as in the commonly used analysis packages and techniques such as SPSS) can lead to underestimation of the strength of the relationships between variables, with reduced factor loadings compared to EFA based upon on the polychoric correlations matrix. Additionally the FACTOR programme provides alternative reliability estimates to the conventional Cronbach’s alpha [45] which, despite its being the most frequently reported measure of internal consistency, has been subject to sustained critical commentary—not simply because it underestimates the reliability of a test and overestimates the first factor saturation, but because it reveals only the *average* degree of “interrelatedness” between scale items, which has a limited relationship to scale internal consistency [46].

Some additional analyses were conducted with SPSS v. 22 [47] using principle axis factoring (PAF) with oblique rotation using the direct Oblimin method, to obtain scree plots for the data, compare EFA findings derived from different procedures and matrices, and known groups scale validation in which differences in R-DAQ sub-scale mean scores were analysed between participant sub-groups (formed on the basis of their involvement in psychiatry education and training), using independent samples *t* tests.

Prior to EFA, the distributions of the R-DAQ items were examined for skewness and kurtosis. Multicollinearity was assessed by examining the tolerance values (which should be close to 1.0) for each item within a linear regression model and the variance inflation factor (VIF) values, which should be less than 10.0, the recommended VIF value to further examine the data for multicollinearity.

The Kaiser–Meyer–Olkin (KMO) measure of sampling adequacy (which may vary between 0 and 1, with values closer to 1 are better, and a value of 0.6 the usual minimum) and Bartlett’s (1954) test of Sphericity (a significant finding enables rejection of the null hypothesis that the correlation matrix is an identity matrix) were used to examine the data for suitability for EFA. The anti-image matrix of correlations was examined to ensure that all elements on the diagonal of the matrix were greater than 0.5, indicating the sample was adequate [48]. Additionally, the suitability of the correlational matrix for factor analysis was checked by examining the number of off-diagonal elements in the anti-image covariance matrix greater than 0.09: the count of off-diagonal elements in the anti-image covariance should be less than 30% [48].

Catell’s scree plot (using SPSS) and parallel analysis [49], a widely recommended Monte Carlo simulation technique (using FACTOR with minimum rank factor analysis and polychoric correlations), were used to determine the number of factors to retain. The adequacy of factor solutions was assessed on the basis the percentage of variance explained, the theoretical coherence of factors, and the simplicity of the factor loadings. Bentler’s [50] simplicity index and the loading simplicity index [51] were used to assess the level of factor simplicity attained in the rotated solution. We tested the goodness of fit of the explanatory model using goodness of fit index (GFI), which ranges between 0 and with values in excess of 0.9 considered an indication of adequate model fit [52], and the root mean square of residuals (RMSR), which was assessed taking into account Kelley’s criterion [53], and also by applying the proposition that if the RMSR is of the order of four divided by the square root of sample size, then a test of significance would not reject the hypothesized model [54].

The reliability and internal consistency of the derived factor solution was examined using a formula based on the standard error of factor scores solutions [55] to provide an estimate reflecting the proportion of variance the items’ factor score accounted for by the underlying common latent variable that drives these item scores, as well as by Cronbach’s coefficient alpha [45], Guttman’s Lambda 6 coefficient (particularly recommended when inter-item correlations are low in relation to squared multiple correlations), and by McDonald’s Omega coefficient (widely considered a more robust estimate of reliability than alpha, based upon square of the correlation between the scale score and the latent variable common to all the indicators in the infinite universe of indicators of which the scale indicators are a subset) [56]. Convergent validity was assessed by performing item-scale correlations corrected for overlaps using Pearson’s product moment correlation coefficient: corrected item-total correlations were considered acceptable if ≥0.2 [57].

## Results

### Respondent characteristics: demographics, training and practice

A total of 601 (85.9%) of the medical practitioners contacted accepted the invitation to participate and completed the survey questionnaires. The frequency of missing values within the returned questionnaires was low: between 0 and 1.5% for R-DAQ items and 0 and 1% for the categorical demographic and related items. The mean age was of the survey participants was 29.7 (SD 7.8), and the male respondents were older (30.5) than females (29.0). Around half (52%) had graduated since 2011, and a similar proportion (52%) were female. Slightly more than half the sample 53.6%) had undertaken some form of psychiatry medical education: either an internship, a psychiatry continuing medical education (CME) programme, or studied psychiatry as a major topic at medical school. The sample characteristics are shown in Table 1: as may be seen, participants who had undertaken additional psychiatry education were more likely to be younger, to have graduated since 2012, to have studied abroad, to have undertaken and CME, and to have ever read a psychiatry article.

**Table 1:** Characteristics of survey participants

Variables	Frequency n (%)		
	Total sample, n = 601	Psychiatry education (intern or major subject, or CME) n = 322	No additional psychiatry education, n = 279
Gender			
Male	286 (47.6%)	155 (48.1%)	131 (47.0%)
Female	315 (52.4%)	167 (51.9%)	148 (53.0%)
Age <28			
<28	321 (53.5%)	185 (57.6%)	136 (48.7%)
Specialty			
Medicine/Paeds	394 (65.6%)	213 (66.1%)	181 (64.9%)
Surgery/ObsGyn	207 (34.4%)	109 (33.9%)	98 (35.1%)
Practice setting			
Rural	78 (13.0%)	52 (16.1%)	26 (9.3%)
Urban	523 (87.0%)	270 (83.9%)	253 (90.7%)
Graduated in 2012 or later (yes/no)			
Yes	311 (52.0%)	184 (57.5%)	127 (45.7%)
Have you ever taken CME courses? (yes/no)			
Yes	298 (49.6%)	172 (53.4%)	126 (45.2%)
Have you studied abroad? (yes/no)			
Yes	102 (17.0%)	71 (22.0%)	31 (11.1%)
Have you ever read an article on psychiatry? (yes/no)			
Yes	202 (33.9%)	142 (44.7%)	60 (21.6%)
How frequently do you encounter depression in your practice setting?			
Seldom	303 (51.1%)	162 (51.3%)	141 (50.9%)
Often	290 (48.9%)	154 (48.7%)	136 (49.1%)

### R-DAQ analysis

#### Descriptives

The distribution of scale response data was examined visually using histograms for each of the 22 items, and multivariate tests for skewness and kurtosis were conducted [58]. There was evidence of some asymmetry: values for skewness were between −1 and −2 for three of the R-DAQ items, and excess kurtosis was evident for four items (for three items this was between −1 and −2; for one it was 2.0). Mardia’s multivariate test for skewness was not statistically significant (test statistic = 4829, *P* = 1.00), but there was evidence of excessive kurtosis (test statistic = 23.4, *P* < 0.001), providing further indication to conduct the factor analysis using the polychoric correlation matrix.

#### Dimensionality

Suitability for EFA was confirmed by the Kaiser–Meyer–Olkin measure of sampling adequacy which was 0.691, and Bartlett’s test of sphericity was statistically significant χ^2^ (231) = 1936.6; *P* < 0.001. The individual measures of sampling adequacy for each of the 22 items were examined in the anti-image of the correlation matrix, and item values ranged between 0.56 and 0.82 and mostly (18/22) in excess of 0.60; the number of off-diagonal elements in the anti-image covariance matrix greater than 0.09 was checked, and the count of off-diagonal elements in the anti-image covariance was 19% (i.e. less than 30%) [48]. Multicollinearity assessed by examination of the tolerance values for each item within a linear regression model showed values between 0.64 and 0.89, and the VIF values of all the variables were less than 1.60.

**Fig. 1:**
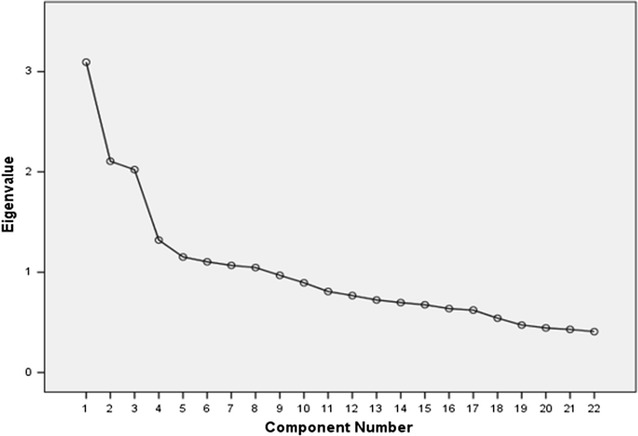
Scree plot, R-DAQ 22-items

Initial EFA showed eight components had eigen values above 1, explaining 58.8% of the variance; but the scree plot (Fig. 1) indicated three components. Parallel analysis (PA) based on minimum rank factor analysis (MRFA) [59] of the 22 item scale likewise indicated three dimensions based on the random explained common variance. The overall percentage of common variance explained, estimated with MRFA for the three factor model, was 49.8%.

Oblique rotation methods are generally recommended for EFA with social science data [60], and Promin rotation (Lorenzo-Seva 1999), the option advised by the developers of FACTOR, was used. Items that fitted poorly with the emergent factor structure in terms of their contribution to the theoretical meaning, or because of weak factor loadings or cross loadings between factors, were successively removed and the analysis repeated. This process led to the removal of seven items: it was apparent for this sample that five items, which in the R-DAQ development process and analysis were indicative of pessimistic or deterministic conceptualisations of depression, fitted poorly for the Pakistani clinicians. Item 9 (*Becoming depressed is a natural part of being old*) strongly cross-loaded with the generalist perspective factor items; whereas items 12 (*Becoming depressed is a way that people with poor stamina deal with life difficulties*), 13 (*Once a person has made up their mind about taking their own life no one can stop them*), 6 (*Depression treatments medicalise unhappiness*) and 5 (*One of the main causes of depression is a lack of self*-*discipline and will*-*power*) exhibited weak and inconsistent loadings.

There was also weak loading for item 19 (*It is rewarding to spend time looking after depressed patients*), whilst item 8 (*I am more comfortable working with physical illness than with mental illnesses like depression*) loaded with the generalist factor rather than (when reversed) fitting with professional confidence in depression management.

The final model for this population was comprised of three factors and contained 15 items (5 items in each of the dimensions). The three factors explained 46.3% of the 15-item scale variance, and these three factors were the same as identified in the R-DAQ development sample: *professional confidence* in depression care, *therapeutic optimism/pessimism* about its course and treatment, and a *generalist perspective* about the occurrence, recognition, and management of depression (Table 2).

**Table 2:** Rotated loading matrix: polychoric correlation, unweighted least squares (ULS), promin rotation

R-DAQ item	Factor		
	Generalist perspective	Professional confidence	Therapeutic optimism
22: Anyone can suffer from depression	0.641*	−0.216	−0.003
2: Depression is a disease like any other (e.g. asthma, diabetes)	0.632*	0.026	−0.162
10: All health professionals should have skills in recognising and managing depression	0.620*	0.164	0.006
14: People with depression have care needs similar to other medical conditions like diabetes, COPD or arthritis	0.593*	0.034	−0.021
16: Recognising and managing depression is often an important part of managing other health problems	0.555*	0.143	0.214
15: My profession is well trained to assist patients with depression	0.018	0.631*	0.082
17: I feel confident in assessing suicide risk in patients presenting with depression	−0.070	0.613*	−0.035
11: My profession is well placed to assist patients with depression	0.063	0.590*	0.045
7: I feel confident in assessing depression in patients	0.104	0.553*	−0.071
1: I feel comfortable in dealing with depressed patients’ needs	0.033	0.417*	−0.012
4: Antidepressant therapy tends to be unsuccessful with people who are depressed (reversed)	−0.102	0.059	0.614*
3: Psychological therapy tends to be unsuccessful with people who are depressed (reversed)	−0.089	0.119	0.545*
20: Becoming depressed is a natural part of adolescence (reversed)	−0.039	−0.102	0.440*
18: Depression reflects a response which is not amenable to change (reversed)	0.095	−0.106	0.392*
21: There is little to be offered to depressed patients who do not respond to initial treatments (reversed)	0.290	−0.139	0.336*

* Factor loading ≥ 0.32

The EFA procedure was also conducted using the principal axis factoring with direct Oblimin rotation, providing an identical structure (the structure matrix is shown in Table 3).

**Table 3:** Structure matrix: Pearson correlation, principal axis factoring, Oblimin rotation (Kaiser normalization)

	Factor		
	Generalist perspective	Professional confidence	Therapeutic optimism
10: All health professionals should have skills in recognising and managing depression	0.621*	0.256	0.055
22: Anyone can suffer from depression	0.594*	−0.083	0.001
2: Depression is a disease like any other (e.g. asthma, diabetes)	0.569*	0.082	−0.068
16: Recognising and managing depression is often an important part of managing other health problems	0.548*	0.262	0.233
14: People with depression have care needs similar to other medical conditions like diabetes, COPD or arthritis.	0.498*	0.137	0.035
15: My profession is well trained to assist patients with depression	0.111	0.620*	0.133
17: I feel confident in assessing suicide risk in patients presenting with depression	0.010	0.581*	−0.003
7: I feel confident in assessing depression in patients	0.169	0.547*	−0.016
11: My profession is well placed to assist patients with depression	0.113	0.545*	0.004
1: I feel comfortable in dealing with depressed patients’ needs	0.072	0.404*	0.032
4: Antidepressant therapy tends to be unsuccessful with people who are depressed (reversed)	−0.034	0.096	0.520*
3: Psychological therapy tends to be unsuccessful with people who are depressed (reversed)	−0.010	0.148	0.475*
20: Becoming depressed is a natural part of adolescence (reversed)	−0.014	−0.046	0.403*
18: Depression reflects a response which is not amenable to change (reversed)	0.092	−0.036	0.357*
21: There is little to be offered to depressed patients who do not respond to initial treatments (reversed)	0.277	−0.025	0.323*

* Factor loading ≥ 0.32

#### Model fit, factorial simplicity and reliability

The measure of fit between this model and the observed covariance matrix was evaluated with the goodness of fit index (GFI) and the value of 0.97 indicated a clearly acceptable model fit. The RMSR value was 0.0575 which likewise indicated the acceptability of the model (as this was only modestly higher than the expected mean value of 0.041 for an acceptable model by Kelley’s criterion; and substantially less than the value 0.163 obtained by applying the criterion involving 4/√sample size). The evaluation of factorial simplicity using Bentler’s simplicity index (S) (0.985) and the loading simplicity index (LS) (0.532) indicated this to be a good structural solution.

The standardised Cronbach’s alpha coefficient calculated for ordinal data for the total (15-items) scale was 0.694, the McDonald’s Omega coefficient was 0.673, and Guttman’s Lambda 6 coefficient was 0.695. The Mislevy and Bock reliability estimate for each of the three factors was also calculated using the FACTOR programme; the reliability of the generalist factor was 0.769, for the professional confidence factor it was 0.732, and for the therapeutic optimism/pessimism factor it was 0.638.

The scale internal consistency based on a Pearson correlation matrix (rather than for ordinal data and using polychoric correlation) measured by Cronbach’s alpha was found to be lower for this population: 0.638 for the total scale, for the *generalist perspective* sub-scale it was 0.706, for the *professional confidence* sub-scale it was 0.667, whilst for the *therapeutic optimism* subscale it was 0.507.

The corrected item-total correlations were examined for the each of the three subscales, and all items exceeded 0.20: the five items in the *generalist perspective* sub-scale were between 0.42 and 0.51; for the *therapeutic optimism* subscale the correlations were between 0.23 and 0.32; and for the *professional confidence* sub-scale the correlations ranged between 0.33 and 0.47.

#### Known groups analysis

Additional validation of the R-DAQ scale within this population was conducted using the known-groups technique, wherein scale responses were compared between two sub-groups based upon an expectation of differing results due to known characteristics. Relationship testing was based on the theoretical assumption that engagement in psychiatry training as a major subject or additional CME, or specialist psychiatry internship, would be associated with greater professional confidence in depression treatment, and increased therapeutic optimism, but lesser endorsement of a generalist perspective.

Modest differences in the expected directions were evident, as shown in Table 4.

**Table 4:** Known-groups R-DAQ subscale scores

Attitude factor sub-scales	Additional psychiatry training, n = 382	No additional psychiatry training, n = 218
	Mean (SD)	Mean (SD)
Generalist perspective (items 2,10,14,16,22)	18.52 (4.14)	19.66 (2.92)
Professional confidence (items 1,7,11,15,17)	16.58 (3.38)	15.21 (3.39)
Therapeutic optimism (items 3,4,18,20,21)	18.36 (3.01)	18.04 (2.67)

Analysis using independent samples *t* test showed statistically significant differences in the hypothesised direction for two of the three R-DAQ subscales: *professional confidence M* = 1.37 (95% C.I. 0.80–1.94), *t* = 4.75, df = 592, P < 0.001; and *generalist perspective M* = −1.14 (95% CI −1.71 to −0.57), *t* = −3.92, df = 569, *P* < 0.001.

The *therapeutic optimism* sub-scale score was higher in the group with additional training and clinical practice, but the difference was not statistically significant: *M* = 0.33 (95% CI −0.16–0.81), *t* = 1.33, df = 587, *P* = 0.18.

## Discussion

### Main findings

This study provides details of the first use of the R-DAQ scale among Pakistani medical practitioners. Psychometric analysis indicates the same three-factor structure as apparent in the predominantly UK-based scale development sample. Item scores for each of the 22 R-DAQ items indicated a less positive perspective about depression and less endorsement of a generalist view about its occurrence and management. The difference in item scores between the Pakistan and UK samples was largest for the pessimistic and deterministic statements that (when reverse scored) comprised the therapeutic optimism sub-scale. These differences were most marked for items 5 (*One of the main causes of depression is a lack of self*-*discipline and will*-*power*), 9 (*Becoming depressed is a natural part of being old*), and item 12 (*Becoming depressed is a way that people with poor stamina deal with life difficulties*). Among this sample of Pakistani doctors, these negative views that depression was related to personal weaknesses and a natural part of growing old were not only widely held, but also appeared to link in the factor analysis with the other scale factors. This necessitated removal of seven items from the scale to provide the most parsimonious solution for its use with this population of clinicians, involving a theoretically sound factor structure and adequate measures of internal consistency and reliability.

The internal consistency for the total scale measured with Cronbach α was lower (0.64, or 0.69 using polychoric matrix and based on 15 items) than obtained within the R-DAQ development sample (0.84). In the current sample, of the three sub-scales, the five item *generalist perspective* sub-scale provided highest reliability (0.77) and internal consistency (0.71) values in contrast to the UK sample where the Cronbach’s α value was 0.57 (total sample) or 0.62 (GPs and adult nurses).

### Strengths and weaknesses

This study was based on a convenience sample of doctors based in six hospitals in Lahore rather than a probability sample representative of the medical staff in Pakistan; this limits generalisations based on these findings, though the selection procedure which sought all of the available medical staff in the selected hospitals, together with the high response rate (86%) provide some indication of the representativeness of the findings. The study was located in the capital of the Punjab and 70% of Pakistani medical students are enrolled in medical colleges in this region, which may add to the generalisability of findings to the wider population. The excess of female doctors (52.4%) in the sample is reflective of the proportion of women (58.7%) registered with the Pakistan medical and dental council [61].

In this study, we did not conduct test-re-test reliability analysis; and our study design did not enable validation of the R-DAQ scale using criterion measures such as (concurrent validity) the extent of correlation with other measures of similar constructs (such as other mental health attitude or stigma scales), or (predictive validity) observations of relevant clinical behaviours (such as extent and quality of depression care delivery, prescribing treatments, or providing referrals). However, in this study, the sample was sufficient to enable a validation procedure using the known groups method, which showed scale variations associated with participant characteristics in accordance with the hypothesised differences.

## Conclusion

Depression is commonly encountered in medical settings and understanding and quantifying the attitudes of general medical staff who play a key role in its identification and treatment is important for examining existing services, determining needs for interventions to improve the quality of care, and for evaluating the effect of approaches designed to improve depression recognition, support and treatment. This relatively large-scale study of medical practitioners in Pakistan has extended the testing of the R-DAQ to a setting and staff group that differ in important ways from that of the scale development study in which the majority of participants were nurses and based in the UK. The psychometric testing has established the content and construct validity of this measure in a Pakistani clinician population, with the same three-factor structure apparent as in the initial study; and it has indicated adequate reliability and internal consistency, albeit for a measure with a reduced number of items.
